# War Charities

**Published:** 1918-03-16

**Authors:** 


### WAR CHARITIES.

### EIGHT THOUSAND NAMES REGISTERED.

The first official list of war charities registered in accordance with the War Charities Act, 1916, and made up to October 21, 1916, contained the names of 953 organisations in England and Wales, fifty-three of which had their headquarters in London. The latest list, which brings the records up to the close of 1917, is an imposing volume of 114 foolscap pages, published by H.M. Stationery Office, and contains not many short of 8,000 titles of institutions and committees, large and small, carrying on charitable work in connection with the war.

The names of several war hospitals occur to us that are not to be found in the list, while others not in any way differing in their character and the source of their authority are present. A number of these hospitals, devoted to the care of discharged sailors and soldiers, hitherto managed by the British Red Cross Society, are now being handed over to the Minister of Pensions, who, in turn, delegates the management to a voluntary hospital or other authority. This process may be the distinctive act of an ordinary or more permanent charity being evolved out of a war charity, and the point in its career when registration under the 1916 Act is no longer necessary.

Some of these charities are things of a moment, such as a Bridge drive or concert in aid of a local hospital or fund, and pass quickly through the list of registrations. Most, however, are more lasting concerns. It is noticed that in many small districts there are to be found quite imposing groups of charities; here is a chance for the organiser to save unavoidable waste effort through a scheme of correlation or affiliation. There is, for example, no apparent reason why the Dorset Comforts Fund and the Dorset T.F. Comfort Fund (Mesopotamia) should not join hands and exercise their kindly offices through one committee.

It is worthy of note that the bodies formed to provide hospitality for Belgian refugees have been reduced in number, and, in view of the many and varied needs that have arisen since 1916, now form but a small proportion of the names in the list. No doubt the bulk of our visitors from across the North Sea have become assimilated in our social and economic scheme, and are proud to find themselves no longer in need of charitable help. But the call upon personal service and financial help, if relieved in one direction, is quickly applied to another, and for every Belgian who claimed our friendship in this country there are now many refugees, Armenian, Roumanian, Serbian, French, or Italian, who, if not here, in other lands, are experiencing keen necessity, to the relief of which the efforts of the charitable are directed.

